# Diffuse large B-cell lymphoma with DNA copy number changes in a Japanese black calf

**DOI:** 10.1007/s11259-024-10371-7

**Published:** 2024-04-05

**Authors:** Masaki Maezawa, Ken-ichi Watanabe, Yoshiyasu Kobayashi, Kio Yoshida, James K. Chambers, Kazuyuki Uchida, Reo Maruyama, Hisashi Inokuma

**Affiliations:** 1 Laboratory of OSG Veterinary Science for Global Disease Management, Graduate School of Agricultural and Life Sciences, The University of Tokyo, Bunkyo-ku, Tokyo, 113-8657 Japan; 2 Project for Cancer Epigenomics, Cancer Institute, Japanese Foundation for Cancer Research, Koto-ku, Tokyo, 135-8550 Japan; 3 Laboratory of Veterinary Pathology, Department of Veterinary Medicine, Inada, Obihiro University of Agriculture and Veterinary Medicine, Obihiro, Hokkaido 080-8555 Japan; 4 Laboratory of Veterinary Pathology, Graduate School of Agricultural and Life Sciences, The University of Tokyo, Bunkyo-ku, Tokyo, 113-8657 Japan; 5 Laboratory of Farm Animal Medicine, Graduate School of Agricultural and Life Sciences, The University of Tokyo, Bunkyo-ku, Tokyo, 113-8657 Japan

**Keywords:** Calf, DLBCL, DNA copy number variation, Japanese Black

## Abstract

A 2-month-old Japanese Black calf exhibited mandibular and superficial cervical lymph node swelling. Fine needle aspiration cytology of the superficial cervical lymph node revealed large lymphoblast-like cells with mitoses. Hematological examination revealed remarkable lymphocytosis with atypical lymphocytes. Increased activities of serum total lactate dehydrogenase and thymidine kinase were detected. At necropsy, generalized swelling of lymph nodes was observed. Histopathological analysis revealed diffuse proliferation of medium-sized round centroblastic neoplastic cells that were positive for CD20, CD79α, PAX5, and BLA-36, and negative for CD3, CD5, CD10, and CD34. The calf was diagnosed with centroblastic diffuse large B-cell lymphoma (DLBCL) based on these findings. Analysis of DNA copy number variation revealed an increased copy number for the *GIMAP* family relative to that in healthy cattle. Moreover, decreases in copy numbers of *GBP-1*, *MIR3141*, *OR5P1E*, and *PTPRG* relative to those in healthy cattle were also observed. Because DNA copy number variation represent a major contribution to the somatic mutation landscapes in human tumors, these findings suggest that DNA copy number changes might have contributed to the onset of DLBCL in the present case.

**Supplementary Information:**

The online version contains supplementary material available at 10.1007/s11259-024-10371-7.

## Introduction

Bovine leukosis/lymphoma is divided into two types: enzootic bovine leukosis (EBL), which is caused by bovine leukemia virus (BLV) infection, and sporadic bovine leukosis/lymphoma (SBL) of unknown cause (Angelos JA et al. 2021). SBL is typically classified into three forms according to onset age and lesion site: calf or juvenile form, thymic form, and cutaneous form. Calf or juvenile SBL is characterized by lymphadenopathy and leukemia in calves. In the thymic form of SBL, the cervical and/or intrathoracic thymus is usually affected in cattle aged 6 to 24 months. In the cutaneous form of SBL, cutaneous plaques can be found on the skin of the entire body in cattle up to 30 months of age. In humans, lymphoma/leukemia is mainly classified according to the World Health Organization (WHO) classification based on a combination of morphology, immunophenotype, genetics, and clinical features (Alaggio et al. 2022). Bovine lymphoma has rarely been classified using the WHO classification.

Although a possible genetic etiology has been suggested for the thymic form of SBL (Parodi et al. 1989), few reports are available on the cause of SBL. Interindividual genetic structural variation is present in many forms, such as single nucleotide polymorphisms and copy number variations (CNVs). CNV is defined as an amplifying or decreasing number of DNA segments 1 kb or larger in the genome (Iafrate et al. 2004; Sebat et al. 2004; Redon et al. 2006), and there is evidence that an increased or decreased copy number can correlate with gene expression levels (Mccarroll et al. 2006; Stranger et al. 2007). In humans, CNV have been associated with onset of several tumors including lymphoma (Lenz et al. 2008; Krepischi et al. 2012; Chapuy et al. 2018). However, to the best of our knowledge, there are no reports on CNV analysis performed in bovine lymphoma. In the present report, we describe a clinical case of diffuse large B-cell lymphoma (DLBCL) with DNA copy number changes in a Japanese Black calf.

## Case presentation

A 2-month-old male Japanese Black calf presented with a chief clinical complaint of superficial masses. At initial examination by a local veterinarian (day 1), enlarged superficial lymph nodes were noted. Hematological examination revealed remarkable lymphocytosis (33,430 /µl; RI: 1,600-5,600/µl) (Dicers and Peel 2018). Bovine lymphoma was suspected by the local veterinarian, and the calf was transferred to the Animal Teaching Hospital at the Obihiro University of Agriculture and Veterinary Medicine to confirm the diagnosis on day 4. On initial physical examination at the hospital, high rectal temperature (40.4 °C; reference interval (RI): 38.0–39.17 °C), tachycardia (102 bpm; RI: 60–84 bpm), and polypnea (84 breaths/min; RI: 18–28 breaths/min) were noted (Dicers and Peel 2018). Swelling of peripheral lymph nodes, including parotid, mandibular, superficial, and subiliac lymph nodes, was observed (Fig. S1). Fine needle aspiration (FNA) cytology of the superficial lymph node revealed the presence of medium to large lymphoblast-like cells with scanty cytoplasm with moderate mitoses (2 mitotic cell per 5 views of 1,000× magnification) (Fig. S2).

In Hospital, hematological examinations revealed lymphocytosis (47,000 /µl), with more than 95% of lymphocytes being morphologically atypical with fine nuclear chromatin and nucleoli, as shown in smear examination of peripheral blood (Fig. S3). Serum biochemical analysis revealed increased activities of total LDH (2,600 U/l; RI: 697–1,450 U/l) and serum thymidine kinase (29.9 U/l; RI: <5.4 U/l) (Ishihara et al. 1980; Sakamoto et al. 2009). LDH isozyme analysis showed elevated activities of LDH-2 (1,045 U/l; RI: 137–503 U/l) and LDH-3 (374 U/l; RI: 82–262 U/l) (Ishihara et al. 1980). Antibodies against BLV were not detected with the BLV Antibody Test ELISA kit (JNC, Tokyo, Japan). Nested polymerase chain reaction (PCR) for the BLV 5′ LTR, which was performed using genomic DNA extracted from peripheral blood samples and swollen superficial cervical lymph node tissue obtained by FNA (Maezawa et al. 2018), yielded negative results.

**Fig. 1:**
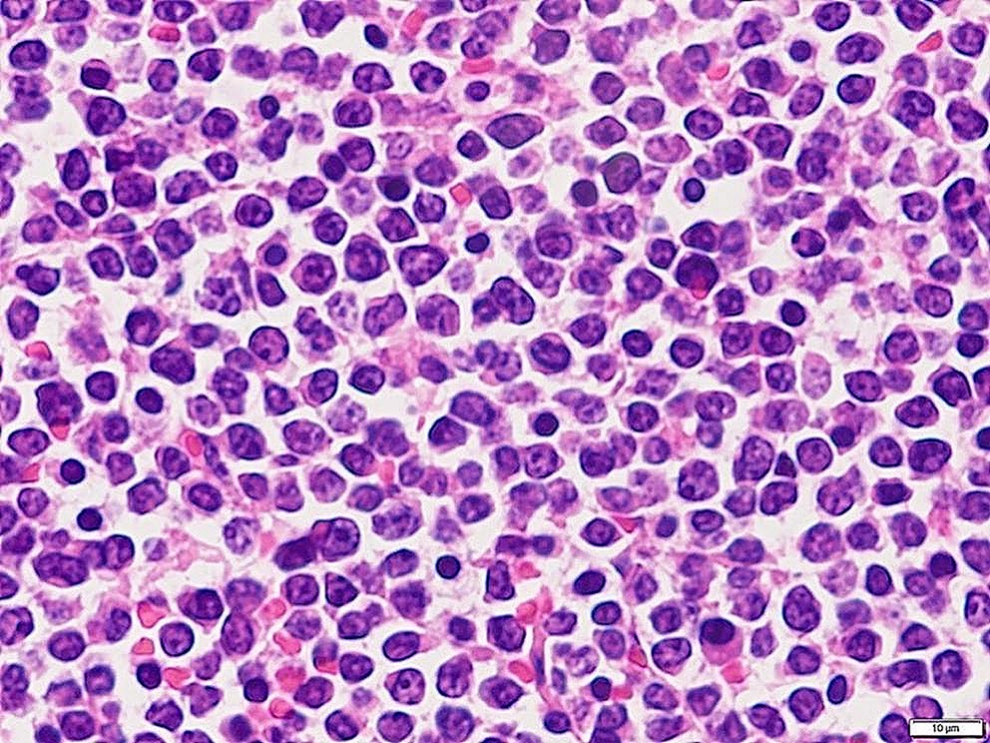
Histopathology of the superficial cervical lymph node. The neoplastic cells were medium to large and round with a small amount of cytoplasm, and showed irregular thickening of nuclear membranes, and crafted nuclei with nuclear atypia. Hematoxylin and eosin stain. Bar = 10 μm

**Fig. 2:**
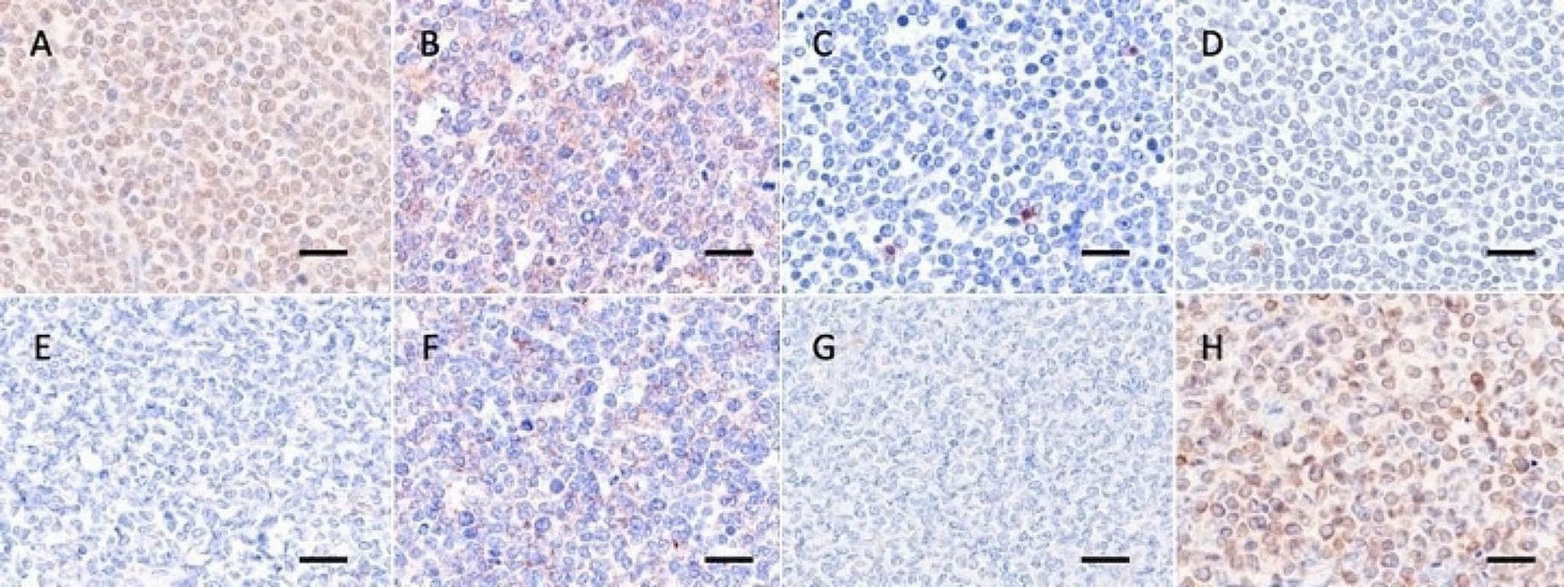
Immunohistochemistry of the superficial cervical lymph node. Histopathological analysis showed that neoplastic cells were positive for B-cell marker (CD20, CD79α, PAX5, and BLA-36) and negative for early lymphohematopoietic stem cells marker (CD10 and CD34) and T cell marker (CD3 and CD5), suggesting tumor cell were mature B cells. (**A**) PAX5, (**B**) BLA-36, (**C**) CD3, (**D**) CD5, (**E**) CD10, (**F**) CD20, (**G**) CD34, and (**H**) CD79α with Mayer’s hematoxylin counterstain. Bar = 20 μm

The animal was euthanized on day 7 for postmortem exam. Grossly, swelling of systemic lymph nodes was observed. A few white foci, 1–3 cm in diameter, were disseminated in the kidneys. The spleen was enlarged (50 × 10 × 3 cm). Histopathological examination revealed infiltration of centroblastic neoplastic lymphoid cells in the enlarged lymph nodes, thymus, bone marrow, spleen, liver, intestines, lung, heart, and kidneys (Fig. 1). Neoplastic cells diffusely proliferated in the affected lymph nodes and organs. The neoplastic cells were medium to large and round with a small amount of cytoplasm, and showed irregular thickening of nuclear membranes, crafted nuclei with nuclear atypia, and granular-patterned chromatin. In immunohistochemical examination, cell membrane of tumor cells within the enlarged lymph nodes immunostained positive for CD20 (Thermo Fisher Scientific, MA, USA) (Inokuma et al. 2023), CD79α (GeneTex, CA, USA) (Lee et al. 2012), and BLA-36 (Biogenex Laboratories, CA, USA) (Maezawa et al. 2022), and negative for CD3 (Dako, CA, USA) (Maezawa et al. 2022), CD5 (Invitrogen, CA, USA) (Okada et al. 2020), CD10 (Diagnostic BioSystems, CA, USA), and CD 34 (Santa Cruz, CA, USA) (Fig. 2 and Fig. S4). Moreover, nucleus cell membrane of tumor cells immunostained positive for PAX5 (BD Biosciences, CA, USA) (Fox et al. 2018).

**Fig. 3:**
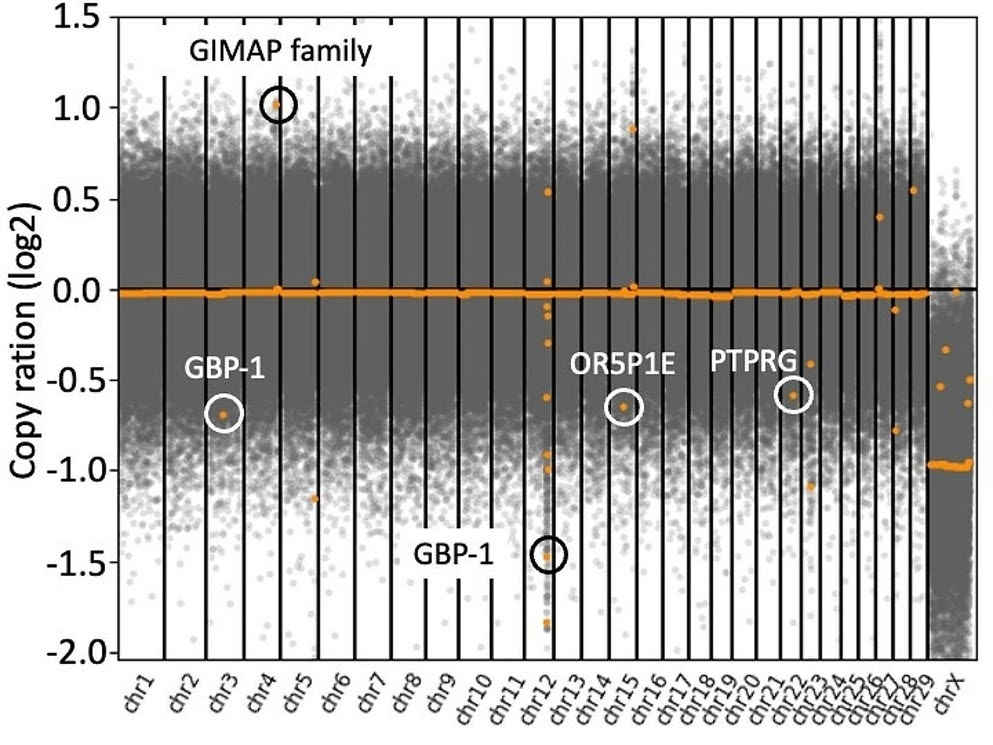
Scatter plot based on the ratio of read counts in the present case to those in healthy cattle. The read counts of most X chromosomal regions in the present calf (male) were half of those in healthy cattle (female). In autosomal regions with transcription units, copy numbers of the coding regions for guanosine triphosphate of the immunity-associated protein family proteins (chromosome 4) in the present calf were more than double those in healthy cattle, and less than half those of the coding regions for guanylate binding protein 1 (chromosome 3), microRNA-3141 (chromosome 12), olfactory receptor family 5 subfamily P member 1E (chromosome 15), and protein tyrosine phosphatase receptor type G (chromosome 22)

CNV was analyzed by CNV sequencing (CNV-seq) using genomic DNA from the neoplastic mesenteric lymph node of the present calf and that from the normal mesenteric lymph node of healthy female Japanese Black cattle. DNA libraries were prepared using a Nextera DNA Flex Kit (Illumina, CA, USA) following the manufacturer’s protocol and sequenced on an Illumina MiSeq system using 2 × 75 bp paired-end reads. Adapter sequences and low-quality bases were trimmed using the Trim Galore script version 0.6.5 (https://www.bioinformatics.babraham.ac.uk/projects/trim_galore/), and trimmed reads (7,240,603 and 9,281,023 reads in the present calf and healthy cattle, respectively) were mapped to the whole genome sequence of *Bos taurus* as provided by UCSC (Bos Tau9) using Bowtie version 2.4.2 (Langmead and Salzberg. 2012). The cattle genome was divided into several continuous regions using 10 kb as the basic unit of analysis, and the number of unique reads that matched in each region was counted using CNVkit (Talevich et al. 2016). The read counts of each region were compared between the present calf and healthy cattle (Fig. 3). The read counts of most X chromosomal regions in the present calf (male) were half of those in healthy cattle (female). In autosomal regions with transcription units, copy numbers of the coding regions for guanosine triphosphate of the immunity-associated protein (GIMAP) family proteins in the present calf were more than double those in healthy cattle, and less than half those of the coding regions for guanylate binding protein 1 (GBP-1), microRNA-3141 (MIR3141), olfactory receptor family 5 subfamily P member 1E (OR5P1E), and protein tyrosine phosphatase receptor type G (PTPRG).

## Discussion

SBL in the present calf was clinically classified as the juvenile form based on age and clinical and molecular findings. Juvenile and thymic forms in SBL is distinguished based on lesion site at grossly level. However, neoplastic infiltration does not always cause enlargement of affected organs (Daiji et al. 2023). In the present case, the thymus was not enlarged on gross exam, although neoplastic cells were observed in the thymus. The presence of neoplastic cells suggested the possibility that enlargement of the thymus due to proliferation of tumor cells might have been observed if the discovery of clinical signs had been delayed. In fact, cases of SBL with enlargement of both the thymus and lymph nodes have been reported (Costa et al. 1992; Hishamnuri et al. 2019). In such cases, distinguishing between juvenile and thymic forms is difficult. Therefore, classification based on lesion site at grossly level might not be very meaningful, and thus, reclassification of SBL might be necessary.

DLBCL is defined as diffuse proliferation of tumorigenic mature B cells in the WHO classification (Alaggio et al. 2022). In the present case, histopathological analysis showed that neoplastic cells were positive for B-cell markers (CD20, CD79α, PAX5, and BLA-36) and negative for early lymphohematopoietic stem cell markers (CD10 and CD34) and T cell markers (CD3 and CD5). These results demonstrated that these centroblastic neoplastic lymphoid cells were not lymphoblastic cell but mature B cells. Moreover, neoplastic cells diffusely proliferated in the affected lymph nodes and tissues. Based on these findings, the present case was diagnosed with centroblastic DLBCL (Valli et al. 2013). More information on immunophenotype in bovine lymphoma/leukemia needs to be accumulated in order to establish a new classification system for bovine lymphoma/leukemia.

Several factors including DNA abnormalities and epigenomic mutations have been reported to play a role in lymphoma development (Galm et al. 2006; Compagno et al. 2009). For instance, *GIMAP* genes are known to regulate lymphocyte development and maintenance (Limoges et al. 2021), and *GIMAP1* and *GIMAP5* are hypomethylated and overexpressed in DLBCL in humans (Chambwe et al. 2014). In the present case, CNV-seq analysis revealed increased copy numbers of *GIMAP* genes, and decreased copy numbers of *GBP-1*, *MIR3141*, *OR5P1E*, and *PTPRG* genes, relative to those in healthy cattle. *GBP-1* mediates anti-pathogenic and anti-proliferative effects in cells and is a gatekeeper of apoptosis and pyroptosis (Itsui et al. 2009; Li et al. 2017; Fisch et al. 2019). *MIR3141* regulates transforming growth factor-β, an important regulator of cell proliferation, differentiation, and apoptosis (Wu et al. 2021). *PTPRG* is involved in the regulation of cell growth, differentiation, mitotic cycle, and oncogenic transformation (Wang et al. 2004; Sorio et al. 1997). Therefore, copy number abnormalities of these genes might have contributed to the onset of lymphoma in the present case. However, the mechanism of copy number changes has been unclear. Further investigation is required to clarify the factors that caused these copy number abnormalities in calf with DLBCL. Moreover, 10 kb is used as the basic unit of analysis in the present case. Additional studies on CNV analysis of more detailed regions are needed.

The present case was diagnosed with DLBCL, and copy number abnormalities of *GIMAP* family genes, *GBP-1*, *MIR3141*, *OR5P1E*, and *PTPRG* were observed. These copy number abnormalities might have contributed to the onset of DLBCL in the present calf, and further investigation in more cattle with DLBCL is needed to elucidate the relationship between CNV and onset of DLBCL in cattle.

### Electronic supplementary material

Below is the link to the electronic supplementary material.

Supplementary Material 1

## Data Availability

No datasets were generated or analysed during the current study.
