# Supplementary material for: Diffuse large B-cell lymphoma with DNA copy number changes in a Japanese black calf
Source: Vet Res Commun. 2024 Apr 5;48(4):2651–6. doi: 10.1007/s11259-024-10371-7 (PMC11315774; doi:10.1007/s11259-024-10371-7)
Supplement: Supplementary file 1 — Supplementary Material 1 [file 11259_2024_10371_MOESM1_ESM.docx]

**
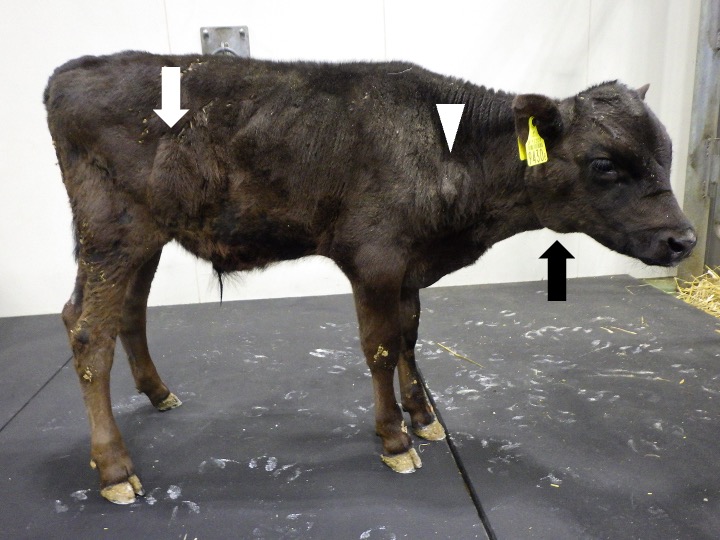
**

**Supplementary Figure 1.** Enlarged superficial lymph nodes were noted on Day 4. White arrow: right subiliac lymph node; white arrowhead: right superficial cervical lymph node; black arrow: right parotid lymph node.


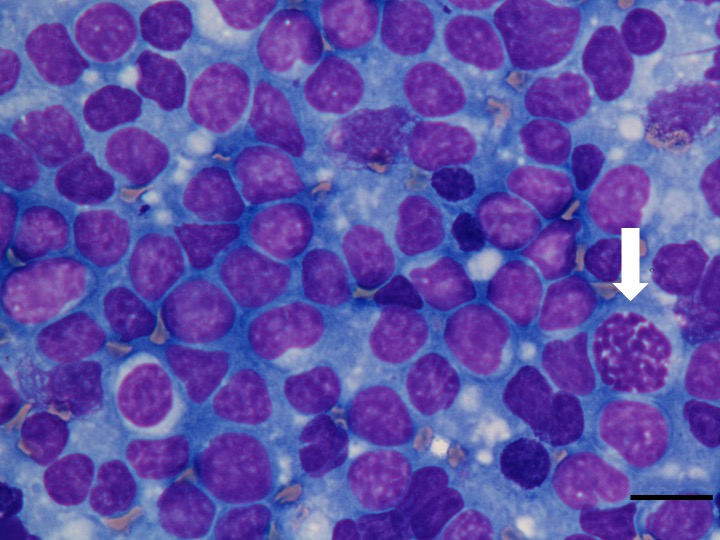


**Supplementary Figure 2.** Fine needle aspiration cytology of the superficial cervical lymph node. The population was composed of medium to large lymphoblasts cells with mitosis. White arrow: mitotic cells. Wright-Giemsa stain. Bar = 10µm.

**
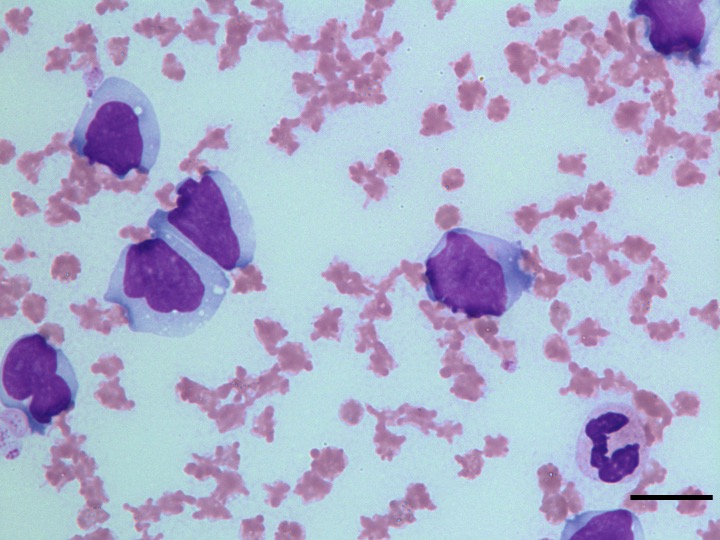
**

**Supplementary Figure 3.** Smear examination of peripheral blood. More than 95% of lymphocytes were morphologically atypical with fine nuclear chromatin and nucleoli. Wright-Giemsa stain. Bar = 10 μm.


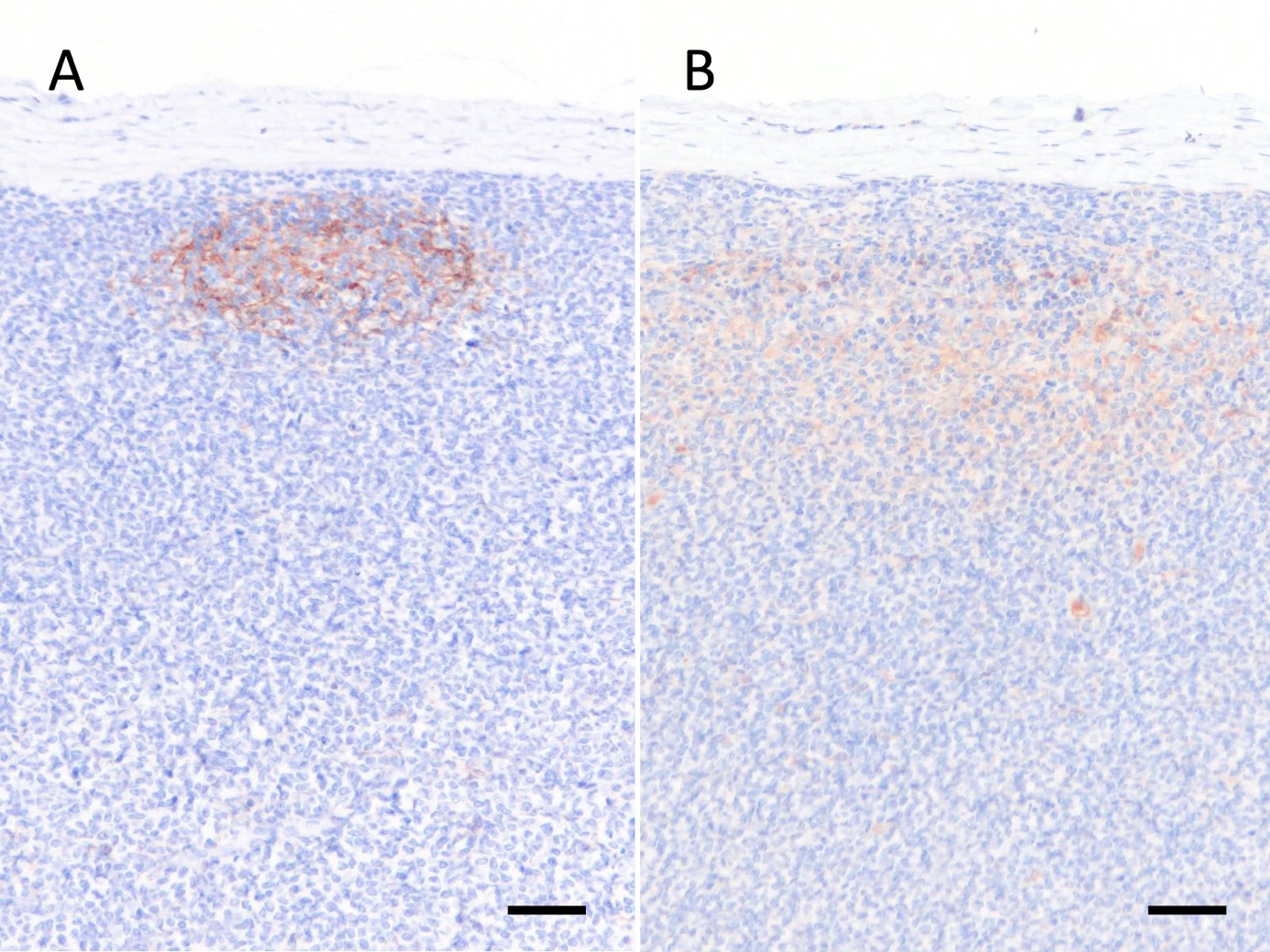


**Supplementary Figure 4.** Immunohistochemistry of CD10 and CD34 controls. (A) and (B): Normal lymph node of the preset case were stained with CD10 and CD34 as positive control. The lymphoid blast cells in lymphoid follicle were stained. Bar = 50 μm.
